# Isoprenaline alleviates diabetic kidney disease via multi-target inhibition of the cGAS–STING pathway

**DOI:** 10.1042/BSR20250174

**Published:** 2026-05-05

**Authors:** Runtao Ma, Yue Song, Lijun Zhang, Peng Wang, Jiaqi Chen, Tianqi Chen, Guangyao Zhu, Qingrong Pan, Mengyu Ma, Qigui Mo, Surui Lu, Xuanjie Yu, Yi Guo

**Affiliations:** 1 School of Basic Medical Sciences, Xianning Medical College, Hubei University of Science and Technology, Xianning 437100, China; 2 School of Pharmacy, Xianning Medical College, Hubei University of Science and Technology, Xianning 437100, China; 3 Radiology, Xianning Central Hospital, The First Affiliated Hospital of Hubei University of Science and Technology, Xianning 437100, China

**Keywords:** cGAS–STING pathway, Diabetic kidney disease, Inflammatory response, Isoprenaline

## Abstract

Diabetic kidney disease (DKD) is a common microvascular complication of diabetes and is closely linked to chronic inflammation. The present study examined how the β-adrenergic receptor agonist isoprenaline (ISO) protected against DKD by regulating the cyclic GMP–AMP synthase–stimulator of interferon genes (cGAS–STING) signaling pathway through multiple targets. In a streptozotocin-induced diabetic mouse model, ISO treatment improved glomerulosclerosis, reduced podocyte injury and proteinuria, and suppressed renal inflammation. Network pharmacology and molecular docking suggested that, besides activating ADRB1/2, ISO may interact with AKT1, SRC, GSK3β, and EGFR, which are involved in cGAS–STING signaling regulation. These findings indicate that ISO alleviates DKD by inhibiting excessive activation of the cGAS–STING pathway through multi-target coordination, providing a new perspective for therapeutic development.

## Introduction

Diabetic kidney disease (DKD) is increasingly recognized as one of the most common and serious complications of diabetes [1–6]. Recent studies suggest that constant inflammation and a malfunctioning immune system are the reasons for DKD [7]. Hyperglycemia-induced mitochondrial damage and oxidative stress trigger sustained release of inflammatory cytokines, such as TNF-α and IL-1β, which in turn exacerbate glomerular cell injury and promote fibrotic signaling [8–10]. Targeting the inflammatory signaling pathways may prove to be an effective means to delay the progression of DKD [11].

The cGAS–STING (cyclic GMP–AMP synthase–stimulator of interferon genes) pathway is an important innate immune sensor of cytosolic DNA. Activation of this pathway is important in DKD-induced inflammatory responses [12,13]. Mitochondrial damage and release of cytosolic DNA due to hyperglycemia or oxidative stress can activate cGAS to produce the second messenger cGAMP [14,15]. cGAMP then activates STING by promoting the phosphorylation of STING mediated by TBK1. STING subsequently activates the downstream transcription factors IRF3 and NF-κB [16], which induce type I interferons and an array of pro-inflammatory cytokines to sustain the chronic low-grade inflammation [17]. Within this context, IRF3 and NF-κB are further modulated by the impact of their signaling molecules AKT1 and GSK3β, which elicit either amplification or negative regulation of STING-mediated signaling [18]. SRC kinase regulates STING phosphorylation and subcellular localization [19], and EGFR contributes to cellular stress-mediated release of inflammatory mediators and tissue repair [20,21]. Collectively, these signaling molecules form a multilayered regulatory network of the cGAS–STING pathway to define the intensity and duration [22].

Isoprenaline (ISO) is a classical non-selective β-adrenergic receptor agonist. Furthermore, it can activate ADRB1/2 receptors to regulate various cellular functions [23]. Previous research has shown that ADRB signaling not only contributes to cardiovascular homeostasis but also curtails inflammation by inhibiting NF-κB and MAPK activity while inducing anti-inflammatory cytokines [24,25]. This suggests that ADRB may play a role in immune modulation. Yet, the impact of ISO in DKD and its underlying molecular mechanism are unclear. ISO may modulate cGAS–STING activity not only through ADRB1/2 activation but also through other key targets, including AKT1, SRC, GSK3β, and EGFR. This indicates the potential of multi-target synergistic modulation of inflammatory responses [26].

**Figure 1:**
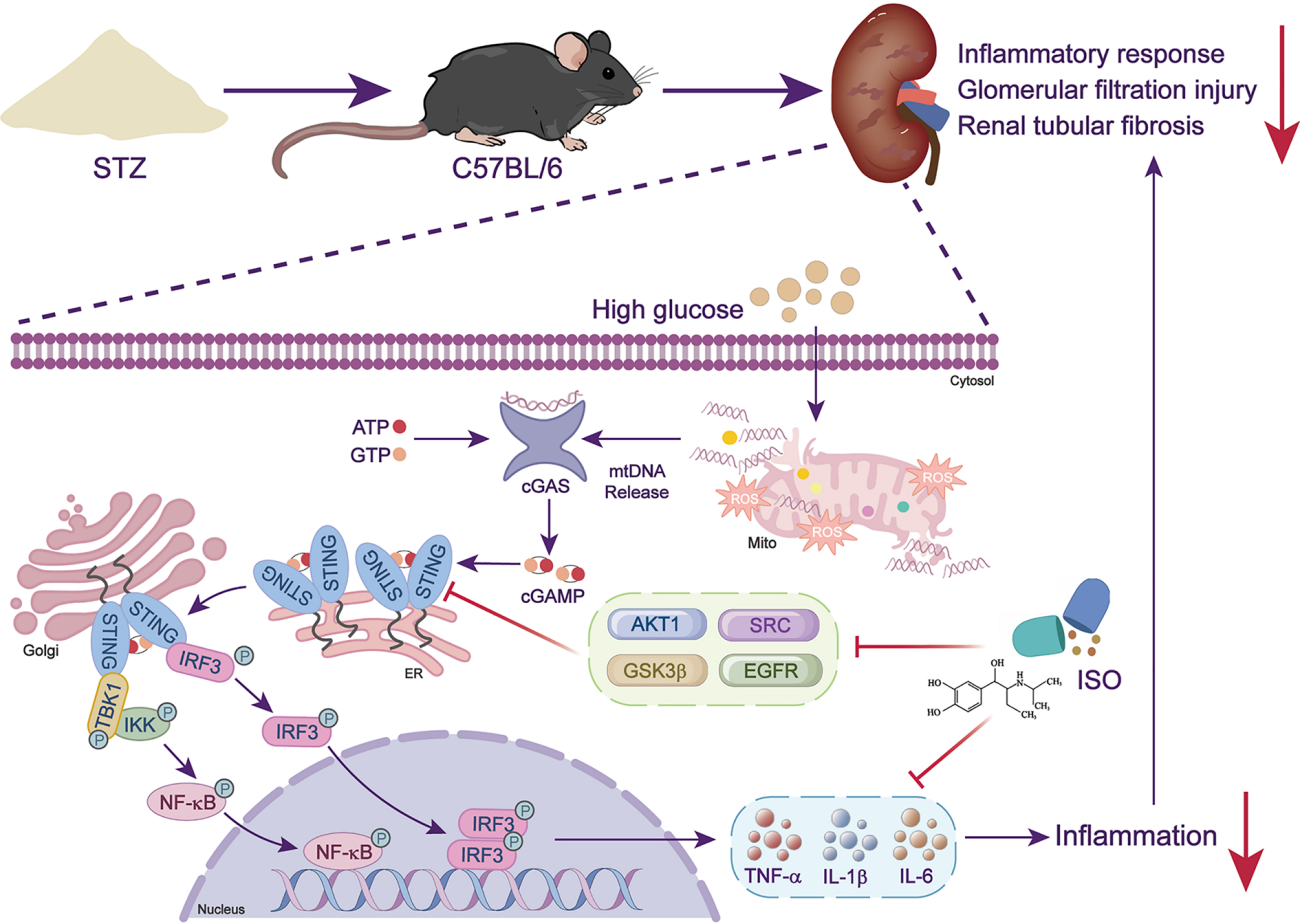
Schematic diagram of the mechanism STZ-induced diabetic C57BL/6 mice exhibit renal pathology characterized by inflammatory cell infiltration, glomerular filtration impairment, and renal tubular fibrosis. Elevated extracellular glucose triggers mitochondrial damage, releasing mitochondrial DNA into the cytosol. This activates cGAS, which synthesizes the second messenger cGAMP to engage STING, thereby initiating IRF3 and NF-κB signaling cascades that drive pro-inflammatory cytokine (TNF-α, IL-1β, IL-6) production. ISO acts through β-adrenergic receptor 1/2 (ADRB1/2) activation and interaction with AKT1, SRC, GSK3β, and EGFR to inhibit cGAS–STING hyperactivation, reducing cytokine secretion and alleviating renal inflammation and tissue injury.

We conducted a detailed investigation on the renal protective effects of ISO in diabetic mice. Our results show that ISO modulates the cGAS–STING signaling pathway by activating ADRB1/2 and potentially interacting with key molecules such as AKT1, SRC, GSK3β, and EGFR to mitigate excessive innate immune activation, renal inflammation and tissue injury (Figure 1). These findings reveal a multi-level molecular mechanism of ISO in DKD, providing a theoretical basis for practical application and development of drugs based on the cGAS–STING pathway in DKD treatment.

## Materials and methods

### Reagents and kits

Streptozotocin (STZ, S10910) was purchased from Aladdin. Isoprenaline (ISO, HY-B0468) and dimethyl sulfoxide (DMSO, HY-Y0320) were obtained from MedChemExpress (MCE). Hematoxylin differentiation solution (G1039), hematoxylin bluing solution (G1040), eosin staining solution (G1004), hematoxylin solution (G1039), phosphate-buffered saline (PBS, G0002), universal tissue fixative (G1101), electron microscopy fixative (G1102), Tween 20 (CR240020), and 5× SDS loading buffer (G2075) were purchased from Servicebio. RIPA lysis buffer (PMK0213), electrophoresis buffer (PMK0202), and transfer buffer (PMK0206) were obtained from Pumei Biotech. Neutral gelatin (BL704A) and other laboratory consumables were from Biosharp. Adhesion microscope slides (188105), standard cover glasses (10212424C), and tissue embedding cassettes (31050102W) were purchased from CITOGLAS. Paraffin (LYHG023054) was from Hualing. Citric acid (10007118) and trisodium citrate (10019418) were obtained from Sinopharm Chemical Reagent. Mouse urine microalbumin (MAU) ELISA kit (E-EL-M0792), creatinine (Cr) colorimetric assay kit (E-BC-K188-M), and urea (BUN) colorimetric assay kit (urease method, E-BC-K183-M) were purchased from Elabscience. Rabbit anti-pSTING S366 (50907), rabbit anti-STING (13647), rabbit anti-pIRF3 S396 (4947), and rabbit anti-pTBK1 S172 (5483) antibodies were obtained from Cell Signaling Technology (CST). Rabbit anti-TBK1 (Ab186470) was purchased from Abcam, and rabbit anti-β-Actin (GB11001) was from Servicebio.

### Animals and model establishment

Male C57BL/6J mice, SPF grade, aged 6–8 weeks with a body weight of 20–25 g, were purchased from Hunan Slack Jingda Laboratory Animal Co., Ltd. Only male mice were used in the present study to minimize variability derived from hormonal fluctuations in female mice, which may interfere with metabolic and inflammatory responses. Mice were housed under SPF conditions with controlled temperature (24 ± 2°C) and humidity (40–70%) in a quiet and well-ventilated environment. All experimental procedures were approved by the Animal Ethics Committee of Hubei University of Science and Technology (Approval ID: HBUST-IACUC-2023-12-209). Animals had free access to food and water and were maintained under a 12-h light/dark cycle. After one week of acclimatization, mice were randomly assigned to either the control group or the diabetic model group. Mice in the diabetic group were fasted and intraperitoneally injected with streptozotocin (STZ, 50 mg/kg, dissolved in citrate buffer, pH 4.4) once daily for five consecutive days. One month after the last injection, fasting blood glucose was measured via tail vein sampling following overnight fasting. Mice with fasting blood glucose ≥11.1 mmol/l were considered successfully modeled for diabetes.

### Animal grouping and management

**Figure 2:**
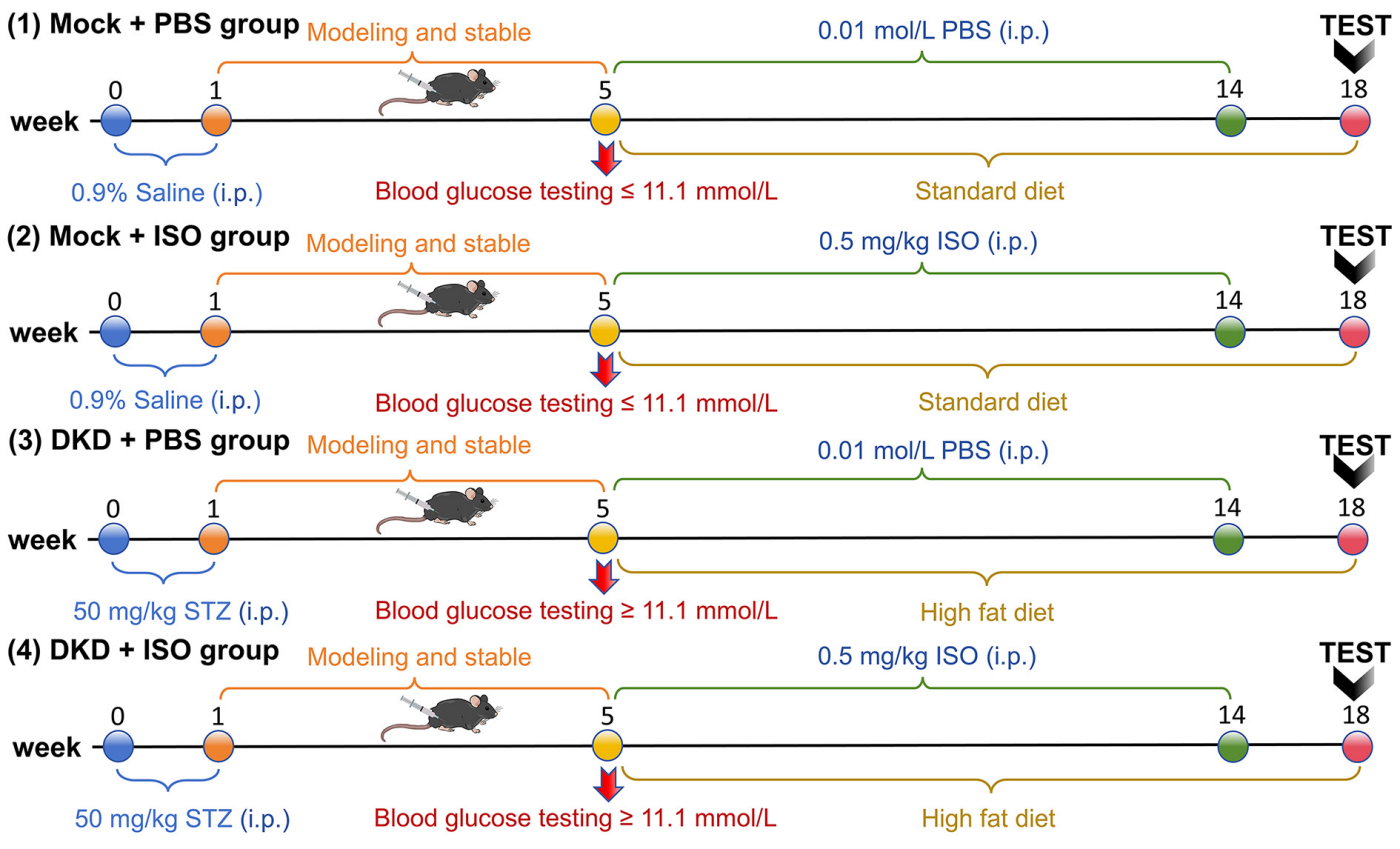
Grouping and experimental schedule Mice were randomized into four groups (*n* = 6 per group): (i) Mock + PBS: Saline-injected control mice fed a standard diet and administered PBS. (ii) Mock + ISO: Saline-injected control mice fed a standard diet and administered isoproterenol (ISO, 0.5 mg/kg/day, i.p.). (iii) DKD + PBS: Streptozotocin (STZ)-induced diabetic mice fed a high-fat diet and administered PBS. (iv) DKD + ISO: STZ-induced diabetic mice fed a high-fat diet and administered ISO (0.5 mg/kg/day, i.p.). After modeling stabilization (verified by blood glucose ≥11.1 mmol/l at week 5), drug interventions were continued for 2 months. Metabolic parameters were analyzed using metabolic cages at week 17, followed by terminal sample collection and endpoint assays at week 18.

After confirmation of DKD induction, mice were randomly assigned to experimental groups. Diabetic mice were divided into two groups: the DKD model group (DKD + PBS) and the DKD + Isoprenaline group (DKD + ISO). Age-matched non-diabetic control mice were randomly allocated into two groups: the Mock group (Mock + PBS) and the Mock + ISO group. A total of four groups were included (*n* = 6 per group). ISO was freshly dissolved in phosphate-buffered saline (PBS) and administered by daily intraperitoneal injection at a dose of 0.5 mg/kg for 9 consecutive weeks. Injections were completed within 30 min of preparation to ensure stability. Mice in the DKD + ISO and Mock + ISO groups received ISO treatment, while mice in the PBS groups received an equal volume of vehicle (PBS). To evaluate sustained effects rather than acute pharmacological responses, animals were observed for an additional 3–4 weeks following the completion of ISO treatment before terminal analyses. The complete experimental timeline is illustrated in Figure 2.

### Sample collection and biochemical analysis

Body weight and fasting blood glucose levels were monitored weekly throughout the experimental period. Diabetic mice were maintained on a high-fat diet (HFD) beginning at weeks 5–6, and HFD feeding was continued until week 18 to sustain metabolic stress. Control mice received a standard diet throughout the study.

At week 17, mice were individually housed in 24-h metabolic cages (Cat. No. DXL-XS, Fengshi Laboratory Equipment Co., Ltd.) for the collection of food intake, water consumption, urine volume, and urine samples. Urinary creatinine and microalbumin levels were measured using commercial ELISA kits (Elabscience) according to the manufacturer’s instructions.

At week 18, blood samples were collected from the retro-orbital sinus and stored at −20°C for subsequent analysis of serum blood urea nitrogen (BUN) and creatinine (Cr). Mice were then anesthetized with 1.5% pentobarbital sodium (Sigma–Aldrich, St. Louis, MO, U.S.A.; intraperitoneal injection) and euthanized by cervical dislocation after confirmation of deep anesthesia. Both kidneys were immediately excised. One portion was fixed in 10% neutral buffered formalin for histological examination, and the remaining tissue was snap-frozen in liquid nitrogen and stored at −80°C for western blotting (WB), quantitative real-time PCR (RT-qPCR), and other molecular analyses.

### Histological staining and analysis

Kidney tissues were fixed at room temperature for 12 h, embedded in paraffin, and sectioned at 4 μm thickness. Images were captured at 400× magnification using an Olympus FV3000 laser scanning confocal microscope with a dry objective lens. Sections were stained with hematoxylin-eosin (HE), periodic acid–Schiff (PAS), and Masson’s trichrome for routine histopathological examination.

For quantitative analysis, six sections per group were evaluated, and eight random fields per section (400×) were analyzed using ImageJ software. Mesangial expansion was calculated as:

$$Glomerular\;Mesangial\;Index\;\left(\%\right)=\frac{Total\;Area\;of\;Glomerular\;Mesangial\;Matrix}{Total\;Area\;of\;the\;Entire\;Glomerulus}\;\times \;100\%$$

Positive staining area was quantified as:

$$PAS-positive\;Area\;Ratio=\frac{Positive\;Area}{\text{Total renal cortex area}}\;\times \;100\%$$

Fibrosis area was determined as the ratio of blue-stained collagen to total tissue area in Masson-stained sections:

$$Masson-positive\;Area\;Ratio\;\left(\%\right)=\frac{Collagen\;Fibers\;Area}{Tissue\;Total\;Area}\;\times \;100\%$$

### Ultra-structural analysis by transmission electron microscopy

Renal cortex tissues were cut into approximately 1 mm^3^ pieces and fixed in 2.5% glutaraldehyde, followed by post-fixation in 1% osmium tetroxide. Samples were dehydrated, embedded, and ultrathin-sectioned for transmission electron microscopy (TEM) analysis (Servicebio, Wuhan, China). Three glomeruli per sample were randomly selected and imaged at 3000×, 7000×, and 20 000× magnifications.

### Network pharmacology analysis of ISO and DKD

The 2D/3D structures and SMILES of ISO were obtained from PubChem (https://pubchem.ncbi.nlm.nih.gov) and uploaded to PharmMapper (http://www.lilab-ecust.cn/pharmmapper/) for human protein target prediction, with gene names converted via UniProt (https://www.uniprot.org/). ISO-related targets were also retrieved from the CTD (http://ctdbase.org/) and SEA (https://sea.bkslab.org/) databases, and duplicates were removed to obtain a non-redundant target list. Venny 2.1 (https://bioinfogp.cnb.csic.es/tools/venny/) was used to generate a Venn diagram to identify overlapping targets between ISO and DKD. The intersecting targets were imported into STRING (https://cn.string-db.org/) to construct a protein–protein interaction (PPI) network, and core targets were screened in Cytoscape 3.9.1 (https://cytoscape.org/) based on degree and centrality parameters. GO (BP, CC, MF) and KEGG pathway enrichment analyses were subsequently performed using Metascape (https://metascape.org/gp/index.html), Using the Microbial Information Online Platform (https://www.bioinformatics.com.cn/), visualize the top 20 significant entries in the form of a bubble chart, with the top 20 significant entries visualized in bubble plots. This analysis systematically integrates ISO potential targets with DKD-related pathways, providing a basis for subsequent experimental validation of key targets and pathways.

### Molecular docking

The 3D structures of target proteins and small-molecule compounds were obtained from the PubChem database (https://pubchem.ncbi.nlm.nih.gov/) and UniProt (https://www.uniprot.org). Molecular docking was performed using the CB-Dock2 online tool to identify the optimal binding conformation with the lowest binding energy [27]. The resulting protein–ligand complexes were visualized and analyzed using PyMOL software.

### Western blot analysis

Renal cortex homogenates were lysed in RIPA buffer and mixed with 5× SDS-loading buffer, vortexed, centrifuged, and denatured at 100°C for 20 min. Fifty micrograms of protein per sample were loaded onto the gels. Proteins were separated by SDS–PAGE and transferred onto PVDF membranes in an ice bath for 1 h at 100 V. Membranes were blocked with blocking buffer containing 5% non-fat milk for 30 min and incubated with primary antibodies at room temperature for 2 h. After washing, membranes were incubated with secondary antibodies for 2 h at room temperature. The signals were developed using an ECL chemiluminescence reagent kit (ECL; Cat. No. 1705060, Bio-Rad), exposed on X-ray film in a darkroom, and then developed and fixed to obtain the images. Band intensities were quantified using ImageJ software.

### Quantitative real-time PCR analysis

Total RNA was extracted from mouse renal cortex using the RNA-Prep Pure Kit and reverse-transcribed into cDNA using the High-Capacity cDNA Reverse Transcription Kit. For cDNA synthesis, 500 ng of total RNA was used per reaction. RT-qPCR was performed on the ABI Prism 7500 system using SYBR Green Master Mix. Relative mRNA expression levels were normalized to GAPDH.

m-TNF qPCR-F (GGTGCCTATGTCTCAGCCTCTT),
m-TNF qPCR-R (GCCATAGAACTGATGAGAGGGAG),
m-IL-1β qPCR-F (GAAATGCCACCTTTTGACAGTG),
m-IL-1β qPCR-R (TGGATGCTCTCATCAGGACAG),
m-IL-10 qPCR-F (CTTACTGACTGGCATGAGGATCA),
m-IL-10 qPCR-R (GCAGCTCTAGGAGCATGTGG),
m-GAPDH qPCR-F (ACGGCCGCATCTTCTTGTGCA),
m-GAPDH qPCR-R (ACGGCCAAATCCGTTCACACC).

### Statistical analysis

Data were presented as mean ± standard deviation (mean ± SD) and analyzed using GraphPad Prism 8 and Excel. Differences between groups were assessed by unpaired t-test or two-way analysis of variance (ANOVA) as appropriate. Statistical significance was indicated as *P <0.05, **P <0.01, and ***P <0.001.

## Results

### ISO alleviates renal stress in diabetic mice

**Figure 3:**
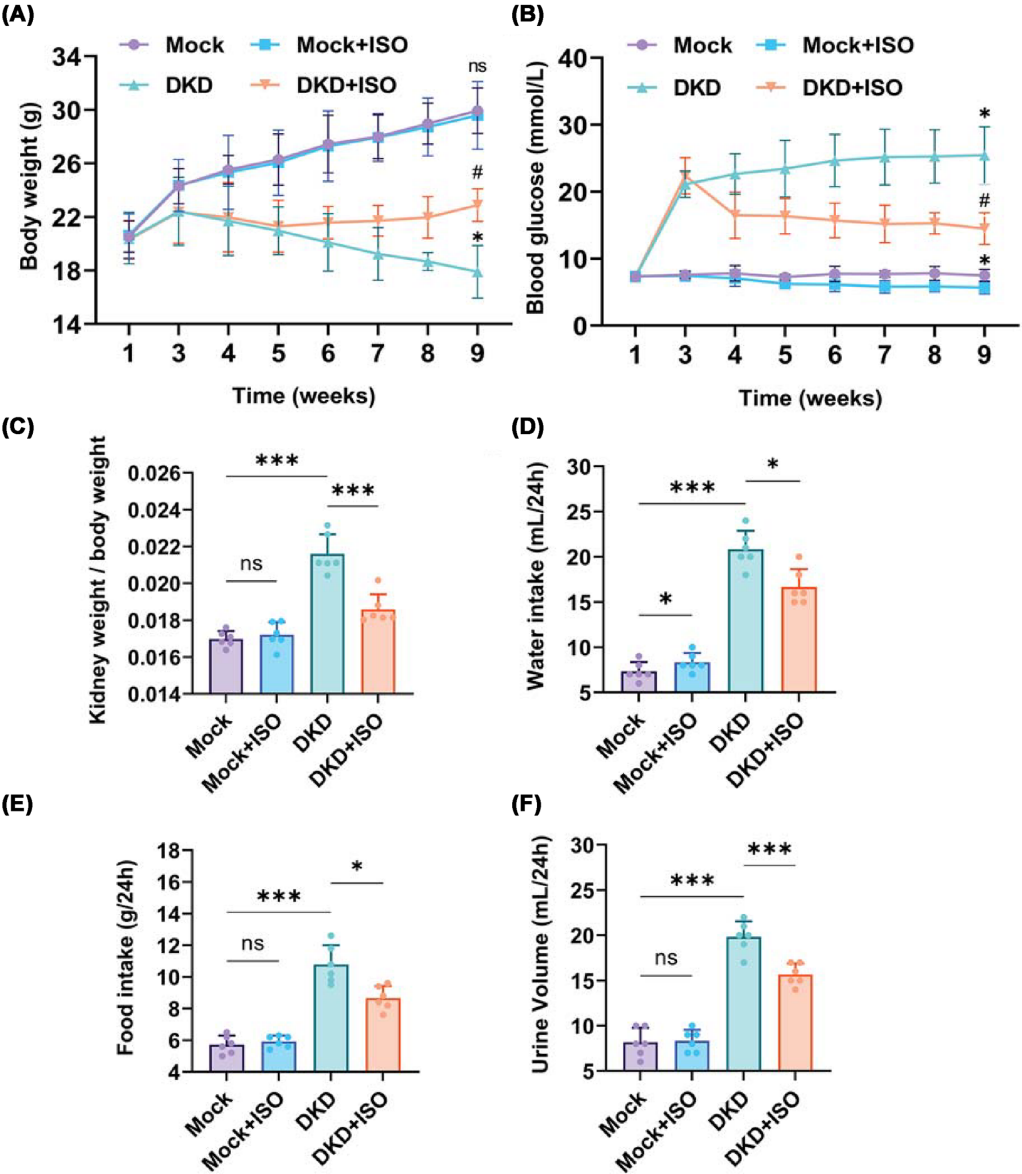
Effects of ISO on body weight, blood glucose, kidney weight, and 24-h metabolic parameters in different groups **(A)** Body weight. **(B)** Blood glucose over 9 weeks. **(C)** Kidney weight/body weight ratio. **(D)** 24-water intake.** (E)** 24-h food intake. **(F)** 24-h urine volume.

**Figure 4:**
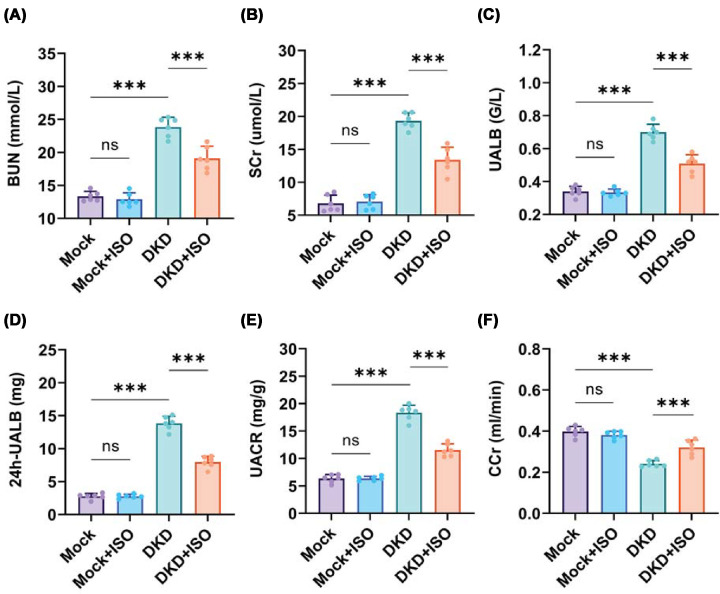
Effects of ISO on renal function markers in different groups (**A**) Blood urea nitrogen (BUN). (**B**) Serum creatinine (SCr). (**C**) Urinary albumin (UALB). (**D**) 24-h urinary albumin (24 h UALB). (**E**) Urinary albumin-to-creatinine ratio (UACR). (**F**) Creatinine clearance rate (Ccr). (*n* = 6. ***P <0.001, ns = not significant).

A diabetes mouse model was established by intraperitoneally injecting male C57BL/6J mice with STZ. ISO was administered to DKD mice in the present study to assess its role in DKD. The Mock + ISO group showed only a slight increase in water intake (Figure 3D) and no significant changes in other parameters compared with the Mock group, indicating that ISO modestly increased metabolic activity without causing significant toxicity in mice. Body weight and fasting blood glucose were measured weekly during the treatment phase after successful model establishment (weeks 5–14, Figure 3A,B). At 17 weeks of age, mice were individually placed in 24-h metabolic cages to record food intake, water consumption, and urine output and to collect urine samples; at this point, mice had been on an HFD for 12 weeks. At 18 weeks of age, body weight and kidney weight were measured during tissue collection to calculate the kidney-to-body weight ratio; at this time, mice had been on HFD for 13 weeks. DKD mice developed typical diabetic symptoms, including body weight loss (Figure 3A), elevated fasting blood glucose (Figure 3B), increased kidney index (Figure 3C), and elevated water intake (Figure 3D), food intake (Figure 3E) and urine volume (Figure 3F). Levels of BUN (Figure 4A), SCr (Figure 4B), UALB (Figure 4C), 24-h UALB (Figure 4D), and UACR (Figure 4E) were significantly elevated, while Ccr (Figure 4F) was decreased, indicating progression to DKD. The relatively lower body weights observed in DKD mice are due to high energy consumption associated with the disease [28–30], which prevents efficient energy storage. Elevated fasting blood glucose, increased water intake, and increased food intake were significantly improved in DKD + ISO mice following ISO treatment (Figure 3), indicating that ISO effectively alleviated the abnormal metabolic phenotypes associated with DKD. Thus, ISO treatment not only effectively alleviated diabetes symptoms but also significantly restored renal function in DKD mice.

### ISO attenuates renal pathology in DKD mice

**Figure 5:**
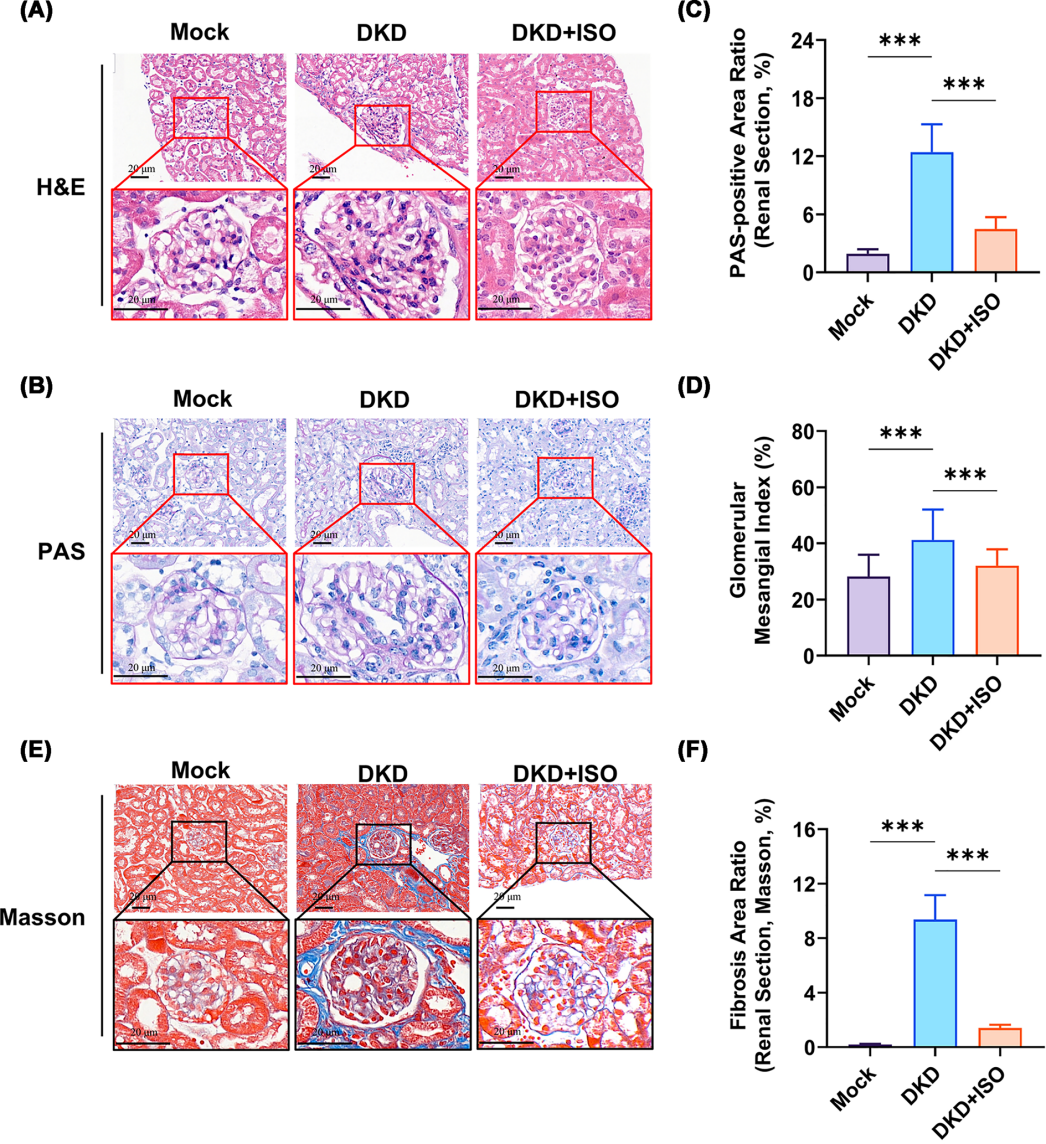
Effects of ISO on kidney histopathology in DKD mice (**A**) Representative H&E staining of renal tissues from different groups. (**B**) Representative PAS staining of renal tissues from different groups. (**C**) Quantification of PAS-positive area in the renal cortex. (**D**) Mesangial matrix index based on PAS staining. (**E**) Representative Masson staining of renal tissues from different groups. (**F**) Quantification of fibrotic area based on Masson staining. (*n* = 6. ***P <0.001).

Since ISO was previously shown to have minimal effects on the Mock group, the Mock + ISO group was not included in the subsequent histological analyses to avoid redundancy and unnecessary use of experimental resources. We conducted HE, PAS, and Masson staining of kidney tissues from each group to assess the protective impact of ISO on renal tissue structure in DKD mice. We assessed overall renal structure and inflammatory injury by using HE staining. We used PAS staining to evaluate mesangial matrix expansion and glycogen deposition. To assess renal fibrosis, we use Masson staining. Mouse kidney tissues were stained with HE (Figure 5A), and the results showed that the glomeruli of the Mock group were normal in morphology and size. In addition, the renal tubules were clear and arranged regularly, and there was no obvious inflammatory cell infiltration. In contrast to control animals, DKD mice displayed excessive glomerular volume, disarray of renal tubules, and significant infiltration of inflammatory cells. The DKD + ISO group showed significant improvement in glomerular morphology and size compared with that in DKD group, while renal tubules were more regularly arranged with significant alleviation in tissue damage. The normal renal tissue structure of Mock group was found intact, as shown from PAS staining in Figure 5B. In contrast, the DKD group exhibited mesangial matrix expansion (Figure 5D). Meanwhile, their renal tubules were found with abundant glycogen, as highlighted in their staining in Figure 5C, which indicated that their condition progressed to DKD. The damage to the renal tissue architecture was lessened by ISO treatment. Masson staining (Figure 5E) showed that compared with Mock, DKD mice showed pronounced fibrosis (Figure 5F). The blue collagen fibers were deposited heavily, primarily in the glomeruli, indicating glomerulosclerosis. Following ISO intervention, the fibrosis level declined significantly, and the collagen depositions reduced to a great extent. This meant ISO treatment alleviated renal structural damage in DKD mice and slowed down the damage process of DKD.

### Effects of ISO on renal ultrastructure in DKD mice

**Figure 6:**
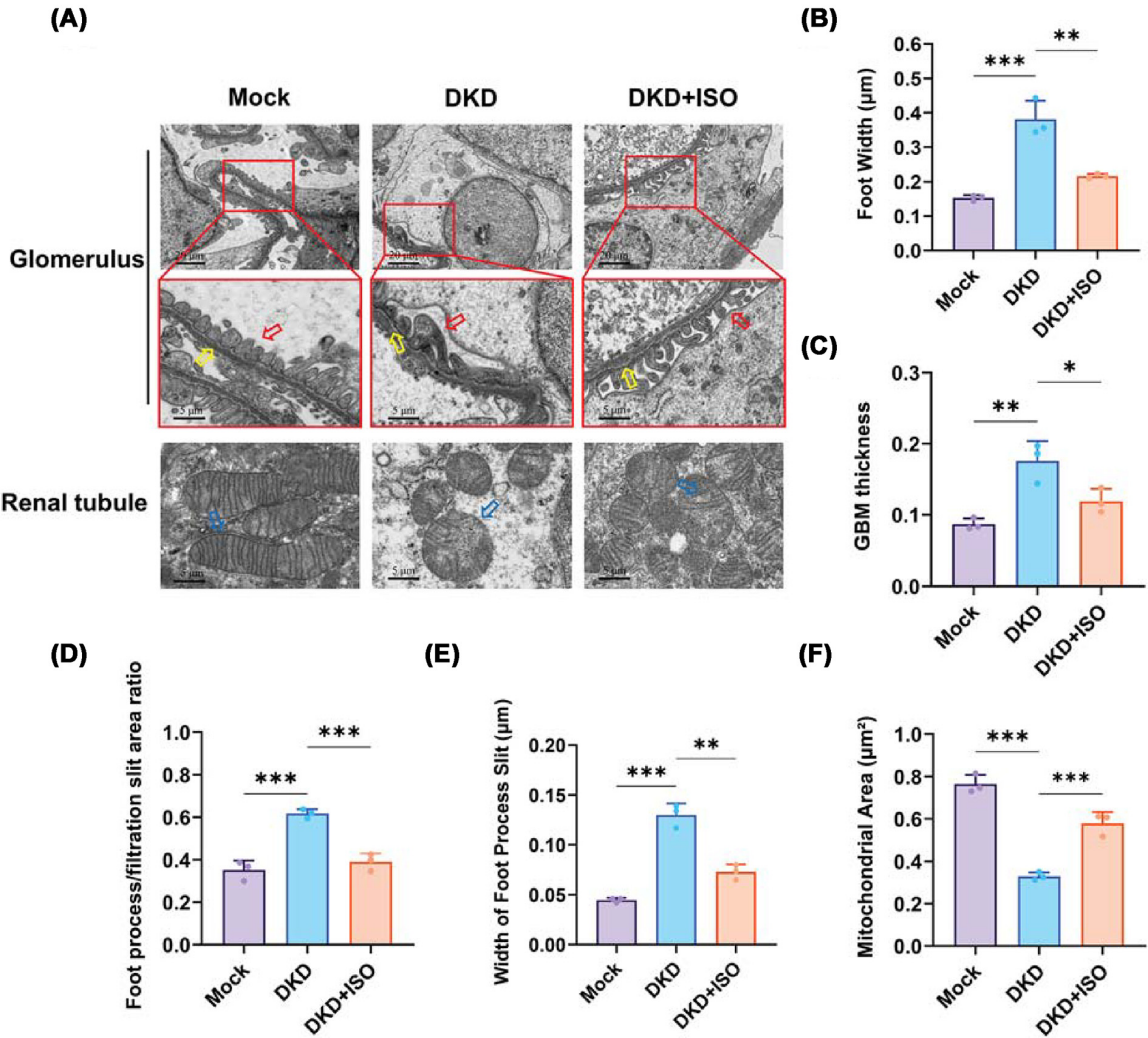
Effects of ISO on renal ultrastructure in DKD mice (**A**) TEM images of renal tissues from different groups. (Red boxes indicate representative observation areas, showing alterations in renal ultrastructure, including glomerular basement membranes and podocyte foot processes. Red arrows indicate foot processes, yellow arrows indicate basement membranes, and blue arrows indicate mitochondrial cristae). (**B**) Quantification of foot process width. (**C**) Quantification of glomerular basement membrane (GBM) thickness. (**D**) Foot process/filtration slit area ratio. (**E**) Quantification of foot process slit width. (**F**) Quantification of mitochondrial area. (*n* = 3. *P <0.05, **P <0.01, ***P <0.001).

TEM has a high-resolution image, which is used to study the ultrastructure of the cell. In this research, the use of TEM was done for the evaluation of ultrastructural damage to podocytes and GBM in diabetic mouse kidney (Figure 6A). The results showed that compared with the Mock group, podocytes of mice with DKD showed widened foot processes (Figure 6B), increased foot process fusion rate (Figure 6D), widened interpodocyte spacing (Figure 6E), and irregular thickening of the GBM (Figure 6C), indicating podocyte injury and glomerular structure abnormalities. Additionally, DKD showed a marked decrease in mitochondrial area (Figure 6F), disrupted cristae, and less dense matrix, indicating impaired mitochondrial function, which may contribute to dysfunction of cellular energy metabolism and increased oxidative stress. These ultrastructural alterations were assessed qualitatively based on established TEM morphological criteria, rather than direct functional measurements. Conversely, these changes at the ultrastructural level were notably reversed by ISO treatment, as indicated by less foot process effacement, increased foot process density, GBM thickness that was largely preserved and comparable to that of the Mock group, and number, structure, and matrix density of mitochondria showing improvement in DKD + ISO groups. The protective effects of ISO described here are therefore based on morphological improvement observed by TEM. These findings suggested that ISO afforded podocyte and GBM protection in diabetic kidneys while mitigating glomerular ultrastructural damage.

### Network pharmacology analysis of ISO in DKD

**Figure 7:**
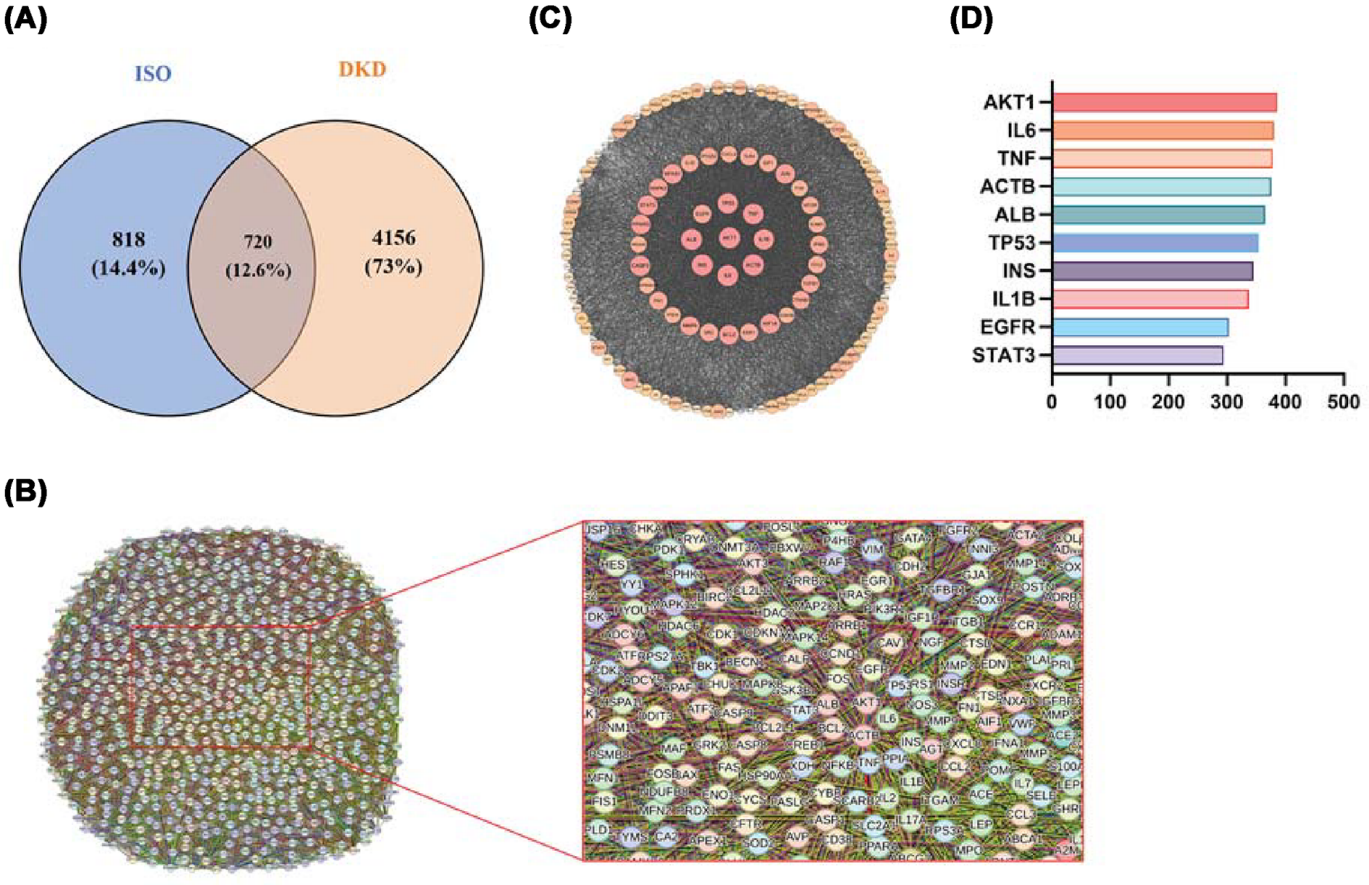
Identification of intersecting targets between ISO and DKD and construction of the PPI network (**A**) Venn diagram showing the overlapping targets shared by ISO and DKD. (**B**) PPI network illustrating the relationships among the active targets of ISO against DKD. (**C**) Visualization of the core target network of ISO in the treatment of DKD. (**D**) Ranking of the top ten hub targets based on degree values.

The PharmMapper, CTD, and SEA databases were used to predict protein targets of ISO. After integrating all data and removing duplicates, 1538 unique protein targets were found. Related genes were gathered using GeneCards, TTD, and OMIM databases. Finally, a total of 4876 nonredundant disease-related genes were identified. The intersection analysis conducted by Venny 2.1 tool resulted in the 720 common targets found between ISO and DKD (Figure 7A). The STRING database was used to import a total of 720 common targets to construct a PPI network of 707 nodes and 23 774 edges (Figure 7B). This network was robust as evidenced by the average node degree (67.3) and a clustering coefficient (0.495). The network was further visualized at Cytoscape 3.9.1 software and the node size and color intensity were positively related to degree value (Figure 7C). The top 10 hub genes were selected based on degree centrality ranking (Supplementary Table S1). These included AKT1, IL6, TNF, ACTB (β-Actin), ALB, TP53, INS, IL1B, EGFR, and STAT3 (Figure 7D). Therefore, these genes could exert major therapeutic effects of ISO against DKD.

**Figure 8:**
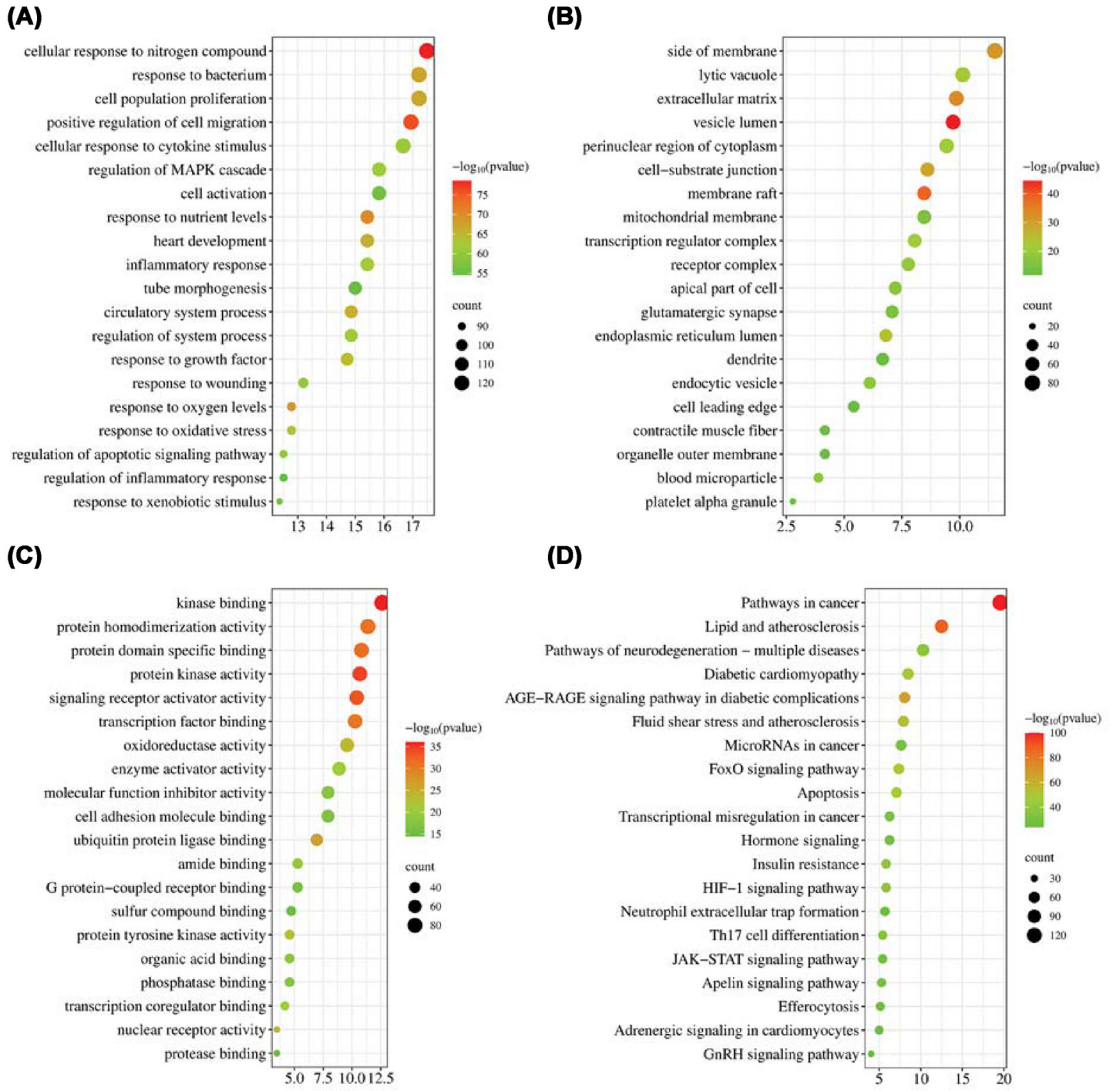
Enrichment characteristics of key network nodes associated with ISO in the treatment of DKD (**A**) Top 20 enriched biological processes (GO-BP). (**B**) Top 20 enriched cellular components (GO-CC). (**C**) Top 20 enriched molecular functions (GO-MF). (**D**) Top 20 enriched KEGG signaling pathways.

GO enrichment analysis revealed that the overlapping ISO–DKD targets were mainly involved in biological processes (BP) of inflammatory response, apoptotic process, and oxidative stress (Figure 8A). Most of the targets in cellular components (CC) were mainly distributed in extracellular space, membrane raft, and receptor complex (Figure 8B). The molecular function (MF) analysis revealed enrichment for enzyme binding, cytokine receptor binding, and identical protein binding (Figure 8C). Analysis of KEGG pathway enrichment (Figure 8D) shows that the targets of ISO were mainly found to be enriched in inflammation, oxidative stress, and metabolism-related signaling pathways, such as AGE–RAGE signaling pathway in diabetic complications, HIF-1 signaling pathway, apoptosis, JAK–STAT signaling pathway, Th17 cell differentiation, and pathways in cancer. Together, these results indicated that the use of ISO may improve kidney function and structural injury during DKD via modulation of these vital pathways and inhibition of cGAS–STING pathway downstream pro-inflammatory and apoptotic signals.

### Molecular docking results of ISO with key target proteins

**Figure 9:**
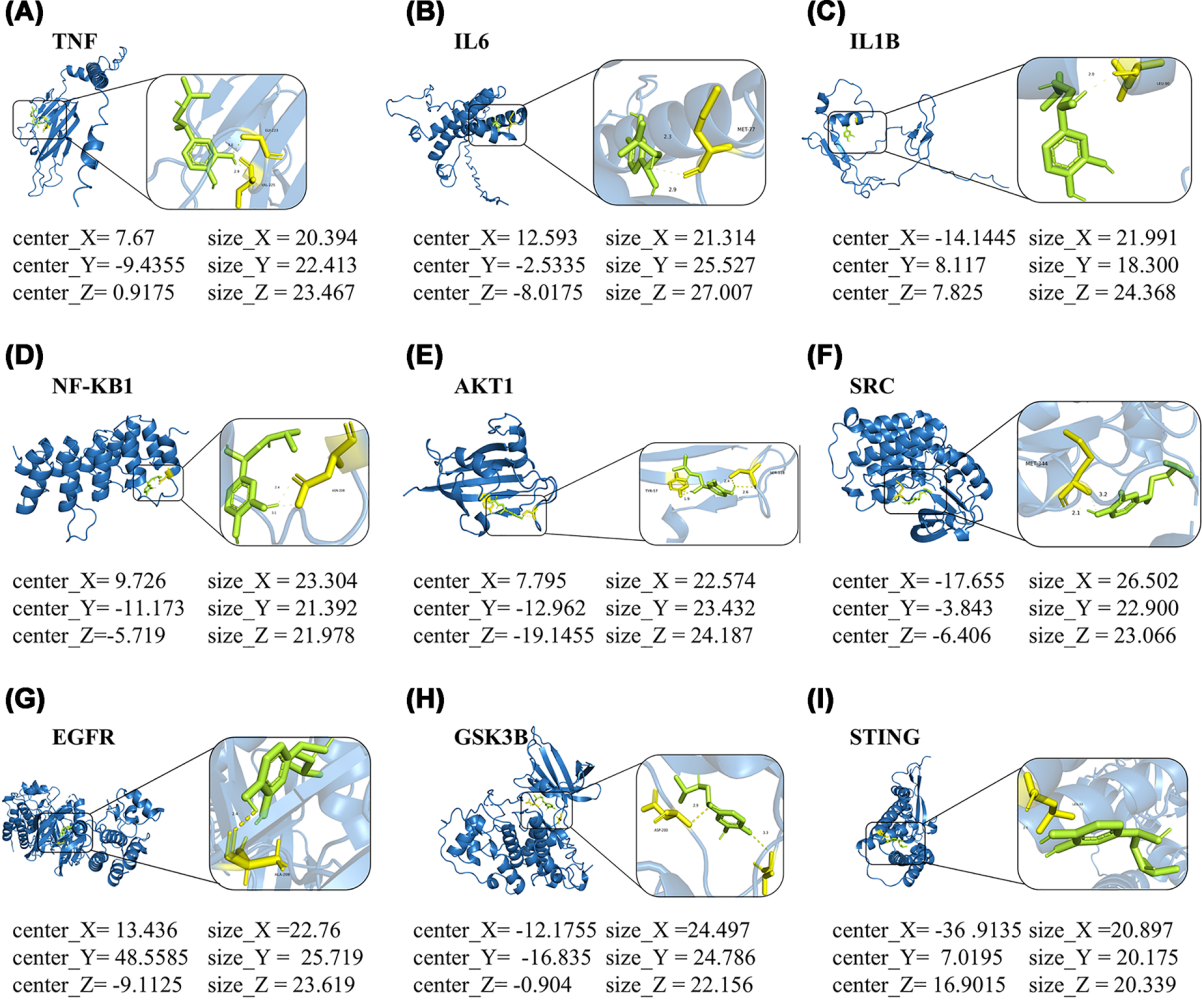
Visualization of molecular docking between ISO and key targets (**A**) Binding model of ISO with TNF (UniProt Entry: C1K3N5). (**B**) Binding model of ISO with IL6 (UniProt Entry: B5MC14). (**C**) Binding model of ISO with IL1B (UniProt Entry: C9JWV2). (**D**) Binding model of ISO with NF-κB1 (UniProt Entry: A0A2K9Y4S6). (**E**) Binding model of ISO with AKT1 (UniProt Entry: 0A087WY56). (**F**) Binding model of ISO with SRC (UniProt Entry: P12931). (**G**) Binding model of ISO with EGFR (UniProt Entry: C9JYS6). (**H**) Binding model of ISO with GSK3B (UniProt Entry: P49841). (**I**) Binding model of ISO with STING (UniProt Entry: A0A494C0W5).

Molecular docking is a technique used in a computer to anticipate the specific binding affinity and mode of interaction of small molecules with target proteins. It is a common tool to understand the interaction of drug candidates and provide a molecular basis for pharmacological studies. A set of 10 core targets associated with DKD was selected based on network pharmacology analysis. These targets were TNF, IL6, IL1B, NF-κB1, AKT1, SRC, EGFR, GSK3B, STING, and ADRB1. Molecular docking was done to evaluate how well ISO can bind and interact with these targets. The docking results (Figure 9 and Supplementary Tables S2 and S3) revealed that ISO possessed binding energies of −5.2 to −7.0 kcal/mol with the 10 core targets, indicating favorable binding stability. The binding energies of AKT1 (Figure 9E), SRC (Figure 9F), EGFR (Figure 9G), and GSK3B (Figure 9H) were less than −5 kcal/mol, indicating stable conformation. The four low-energy targets were involved in cGAS–STING signaling pathway regulation. This underscored the importance of genes in inflammation and cell repair. The results implied that ISO may interact specifically with these core targets to modulate the activity of the cGAS–STING pathway, before alleviating inflammation, improving mitochondrial function, and promoting organ/tissue repair. Moreover, further molecular docking analysis of ISO with STING (Figure 9I) and its endogenous positive receptor ADRB1 (Supplementary Figure S1) substantiated that ISO might exert a multi-target interaction to modulate DKD-related signaling pathways and the reliability of the above docking results.

### Experimental validation of ISO effects on the cGAS–STING signaling pathway and inflammatory response in DKD mice

**Figure 10:**
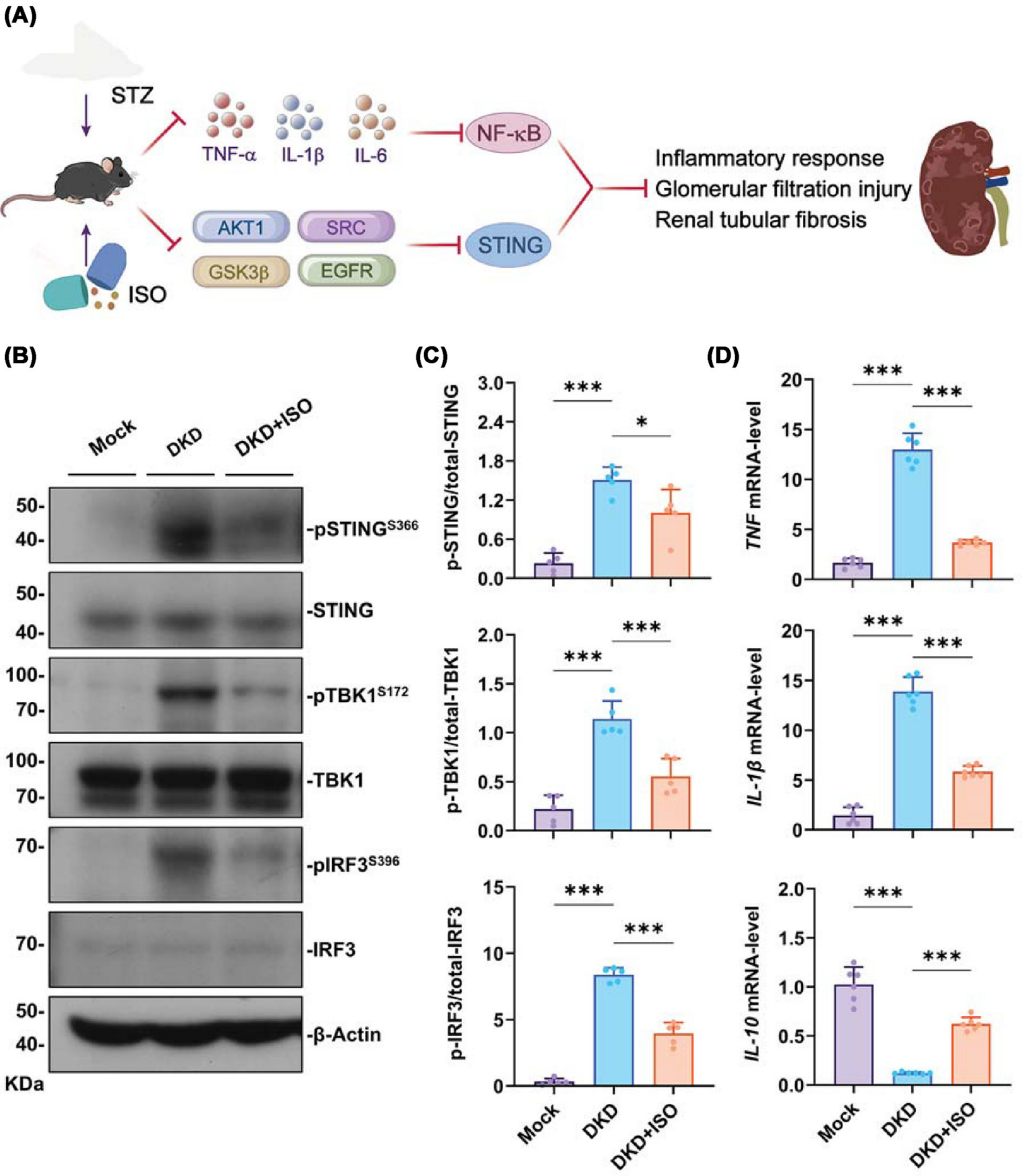
Effects of ISO on key protein expression and inflammation-related indicators in the renal tissue of diabetic mice (**A**) Schematic illustration of the cGAS–STING signaling pathway and its role in renal inflammation. (**B**) Western blot analysis showing the protein levels of p-STING^S366^, STING, p-TBK1^S172^, TBK1, p-IRF3^S396^, IRF3, and β-Actin in Mock, DKD, and DKD + ISO groups. (**C**) Quantification of the relative phosphorylation levels: p-STING^S366^/STING, p-TBK1^S172^/TBK1, and p-IRF3^S396^/IRF3. (**D**) mRNA expression levels of *TNF*, *IL-1β*, and *IL-10* in renal tissues. (*n* = 5–6. *P <0.05, ***P <0.001).

To determine whether ISO regulated DKD through the cGAS–STING signaling pathway (Figure 10A), western blot analysis was carried out. The results shown in Figure 10B,C revealed that compared with the Mock group, the DKD group had significantly elevated phosphorylated STING (p-STING^S366^), TBK1 (p-TBK1^S172^), and IRF3 (p-IRF3^S396^) in kidney tissue, thereby demonstrating activation of cGAS–STING signaling pathway. In the DKD + ISO group, the phosphorylation levels of STING, TBK1, and IRF3 were significantly reduced compared with that of the DKD group (Figure 10B,C). This suggested ISO can inhibit the hyperactivation of the STING pathway. Moreover, assessment of inflammation-related gene expression (Figure 10D) revealed that DKD mice had reduced levels of IL-10, an anti-inflammatory cytokine, and increased levels of IL-1β and TNF, which are pro-inflammatory cytokines, when compared with the Mock group. The anti-inflammatory effects of ISO treatment were indicated by the rescued transcription levels of these genes towards normal. The overall results suggested that ISO could reduce the inflammatory response of DKD possibly through inhibiting cGAS–STING signaling pathway along with its downstream pro-inflammatory mediators.

## Discussion

In the present study, we systematically examined the mechanisms responsible for the action of ISO in a DKD mouse model, especially the modulations of cGAS–STING signaling by various molecular targets. ISO is a non-selective β-adrenergic receptor agonist that activates ADRB1/2 to influence various cellular processes [31], yet the precise role against DKD has yet to be fully elucidated.

In DKD mice, ISO showed protective effects at the systemic level and renal level. ISO therapy reduced body weight loss and hyperglycemia, causing a decrease in kidney-to-body weight ratio and ameliorating renal function indices such as BUN, SCr, UALB, UACR, and CCr. The findings suggest that ISO can counteract metabolic disturbances induced by diabetes such as renal dysfunction. Their results indicate that protection of the kidney by ISO may be due to metabolic homeostasis at least in part [32].

Histological tests also showed excellent impact of ISO on renal inflammation, mesangial expansion, and interstitial fibrosis. This means that ISO may curtail chronic inflammation and inhibit fibrotic signaling, thereby delaying degenerative structural changes in the kidney. The progression of DKD is significantly affected by the inflammatory response and immune imbalance [33]. Mesangial proliferation occurs closely linked with TGF-β/Smad signaling and NF-κB signaling [34]. Improvement in tissue pathology by ISO may reduce pro-inflammatory cytokine release, inhibit mesangial proliferation, and reduce collagen deposition, providing multi-layer renal protection.

Ultrastructural evaluation demonstrated the cytoprotective effects of ISO. Treatment with ISO led to improved morphology of podocyte foot processes. GBM thickness was restored toward normal levels. The number and structure of mitochondria were restored. These data imply that ISO alleviates diabetes-induced oxidative stress and dysfunction of energy metabolism and protects podocytes from chronic inflammation and hyperglycemic damage. Podocyte foot process and GBM integrity are crucial for glomerular filtration barrier function [35–37]. Furthermore, better mitochondrial function may further reduce ROS generation and downstream activation of pro-inflammatory signaling and thus offer a mechanism for the attenuation of glomerular injury [38–40].

The collective findings from network pharmacology, molecular docking, and western blot analyses suggested that ISO may inhibit the DKD-related chronic inflammation process through regulating the cGAS–STING pathway. ISO demonstrated low interaction energies with AKT1, SRC, GSK3β, and EGFR, indicating stable interactions and possible regulation of signaling. AKT1 and GSK3β function as important downstream regulators of cGAS–STING by altering IRF3 activation and NF-κB transcription [41]. On the other hand, SRC and EGFR regulate STING phosphorylation and subcellular localization to amplify or attenuate inflammatory signaling [42,43]. Thus, ISO most likely exerts multiple-layered control over cGAS–STING through various target engagement rather than single-target inhibition. The wide-ranging target interaction results in improving resilience against inflammatory responses and reducing the compensatory effects associated with individual-target agents. Also, it provides more stable therapeutic effects.

Western blot analysis revealed that ISO markedly inhibited phosphorylation of STING, TBK1, and IRF3 [44]. restored IL-10 expression, and suppressed IL-1β and TNF-α induction. The findings suggest that ISO controls cGAS–STING’s upstream activation and downstream transcription activation simultaneously. This ensures that a coordinated co-expression of pro-inflammatory and anti-inflammatory gene activation occurs [45–47]. Subsequently, through inhibition of AKT1, SRC, GSK3β, and EGFR, ISO limits pro-inflammatory signaling while possibly enhancing cellular repair and antioxidant capacity [48,49]. As a whole, it modulates the renal microenvironment.

KEGG pathway analysis to determine whether the ISO-associated target genes were enriched in the AGE–RAGE pathway, HIF-1 pathway, JAK–STAT pathway, Th17 differentiation pathway, and apoptosis pathway. All of these pathways are closely related to cGAS–STING-mediated inflammatory signaling [50,51]. It can be anticipated that ISO multi-target modulation can simultaneously ameliorate inflammation, apoptosis, oxidative stress, and hospital protection against chronic disease progression. Given that complex metabolic disorders generally exhibit functional disruptions of connected pathways, implementing agents capable of multi-target activity, much like ISO, can help regain network homeostasis while minimizing maladaptive compensatory responses and improving therapeutic impact and mechanistic stability [52].

## Conclusion

The present study evaluated the protective effects of ISO in a DKD mouse model. Our results demonstrated that ISO improved metabolic disturbances and renal dysfunction, reduced renal inflammation and fibrosis, and alleviated podocyte and GBM damage. Mechanistically, ISO may influence the cGAS–STING pathway and its downstream inflammatory signaling, potentially involving key molecules such as AKT1, SRC, GSK3β, and EGFR. Further studies are needed to directly assess how ISO affects the activation and function of these proteins, which could provide deeper mechanistic insight. Overall, these findings provide experimental support for the protective effects of ISO in DKD and lay the groundwork for future investigations into its underlying mechanisms.

## Supplementary Material

Supplementary Figure S1 and Tables S1-S3

## Data Availability

The data that support the findings of the present study are available from the corresponding author upon reasonable request. All relevant data generated or analyzed during the present study are included in this published article.

## Abbreviations

ADRB: β-adrenergic receptor
ALB: albumin
BP: biological processes
BUN: blood urea nitrogen
CC: cellular components
cGAMP: cyclic GMP-AMP
cGAS: cyclic GMP-AMP synthase
Cr: creatinine
DKD: diabetic kidney disease
EGFR: epidermal growth factor receptor
GBM: glomerular basement membrane
GSK3β: glycogen synthase kinase 3 beta
IL: interleukin
IRF3: interferon regulatory factor 3
ISO: isoprenaline
JAK: janus kinase
MCP: monocyte chemoattractant protein
MF: molecular function
OMIM: online mendelian inheritance in man
ROS: reactive oxygen species
RT-qPCR: quantitative real-time PCR
SEA: similarity ensemble approach database
STING: stimulator of interferon genes
STZ: streptozotocin
TEM: transmission electron microscopy
TGF-β: transforming growth factor beta
TNF: tumor necrosis factor
TTD: therapeutic target database
UACR: urine albumin-to-creatinine ratio
