# Supplementary material for: Isoprenaline alleviates diabetic kidney disease via multi-target inhibition of the cGAS–STING pathway
Source: Biosci Rep. 2026 May 5;46(5):BSR20250174. doi: 10.1042/BSR20250174 (PMC13161330; doi:10.1042/BSR20250174)
Supplement: Supplementary Figure S1 and Tables S1-S3 [file BSR-2025-0174_supp.pdf]

ADRB1

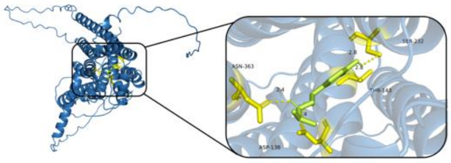

center_X= 1.0825	size_X= 26.999
center_Y= 5.8425	size_Y= 24.391
center_Z= -11.43	size_Z= 22.636

Figure S1 Binding diagram of ISO with ADRB1 (Entry ID: P08588 in the UniProt database).

Table S1 **Top 40 targets ranked by degree value among the intersection targets of ISO and diabetic kidney disease (DKD).**

Name	Degree
AKT1	386
IL6	380
TNF	378
ACTB	375
ALB	365
TP53	354
INS	345
IL1B	337
EGFR	303
STAT3	294
CTNNB1	291
CASP3	277
HIF1A	277
MYC	272
JUN	270
SRC	269
BCL2	269
MMP9	264
NFKB1	262
FN1	261
IFNG	255
MAPK3	254
TGFB1	253
ESR1	250
IL10	246
PPARG	243
CCL2	238
TLR4	238
HSP90AA1	229
IGF1	220
CXCL8	216
PTGS2	216
PTEN	213
CCND1	208
FOS	206
HSP90AB1	205
AGT	200
ICAM1	197
MTOR	196
GSK3B	195

Table S2 The quantitative results of molecular docking binding energy between the active component ISO and diabetes-related key target proteins (including TNF, IL6, IL1B, NF-KB1, AKT1, SRC, EGFR, GSK3B, STING, and ADRB1), with the unit being kilo-calories per mole (kcal/mol).

Name	Binding energy (kcal/mol)
TNF	-6.3
IL6	-6.2
IL1B	-5.2
NF-KB1	-5.9
AKT1	-6.3
SRC	-6.3
EGFR	-6.7
GSK3B	-5.9
STING	-5.6
ADRB1	-7.0

Table S3 Results of molecular docking root mean square deviation (RMSD) between ISO and key target proteins. The RMSD values (unit: angstrom, Å) represent the structural deviation between the docked complex conformations formed by ISO and each target (TNF, IL6, IL1B, NF-KB1, AKT1, SRC, EGFR, GSK3B, STING, ADRB1) and their respective reference conformations. A smaller RMSD value indicates higher consistency between the docked conformation and the reference state, thereby suggesting more reliable molecular docking results.

Molecular docking content	RMSD(Å)
ISO-TNF	1.398
ISO-IL6	2.455
ISO-IL1B	1.963
ISO-NF-KB1	2.740
ISO-AKT1	2.614
ISO-SRC	2.647
ISO-EGFR	2.391
ISO-GSK3B	2.683
ISO-STING	2.137
ISO-ADRB1	1.998

Figure 10B

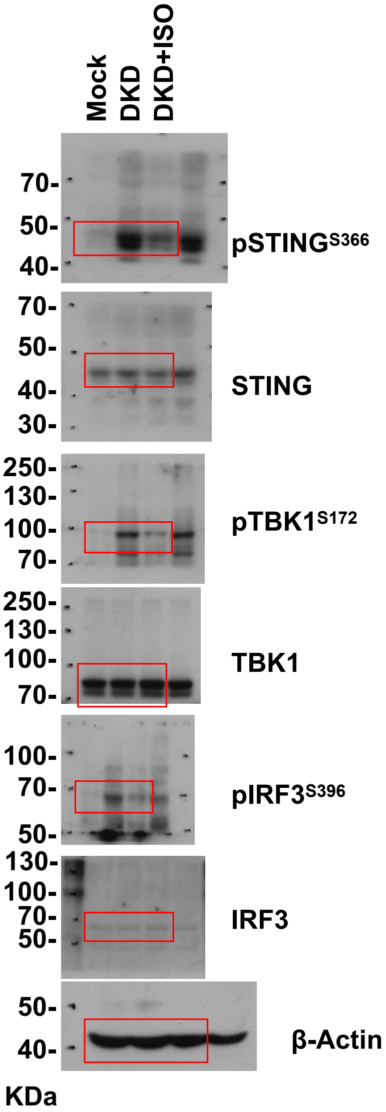

The signals were developed using an ECL chemiluminescence reagent kit, exposed on X-ray film in a darkroom, and then processed using a DL-series automatic film processor. The standard development cycle included a 90-second development at 35°C, followed by fixing, washing, and drying to obtain the images.

These images include all western blot results shown in Figure 10B. The groups selected for presentation are indicated by red boxes, and the corresponding original images with clearly visible membrane edges are provided on pages 2–8.

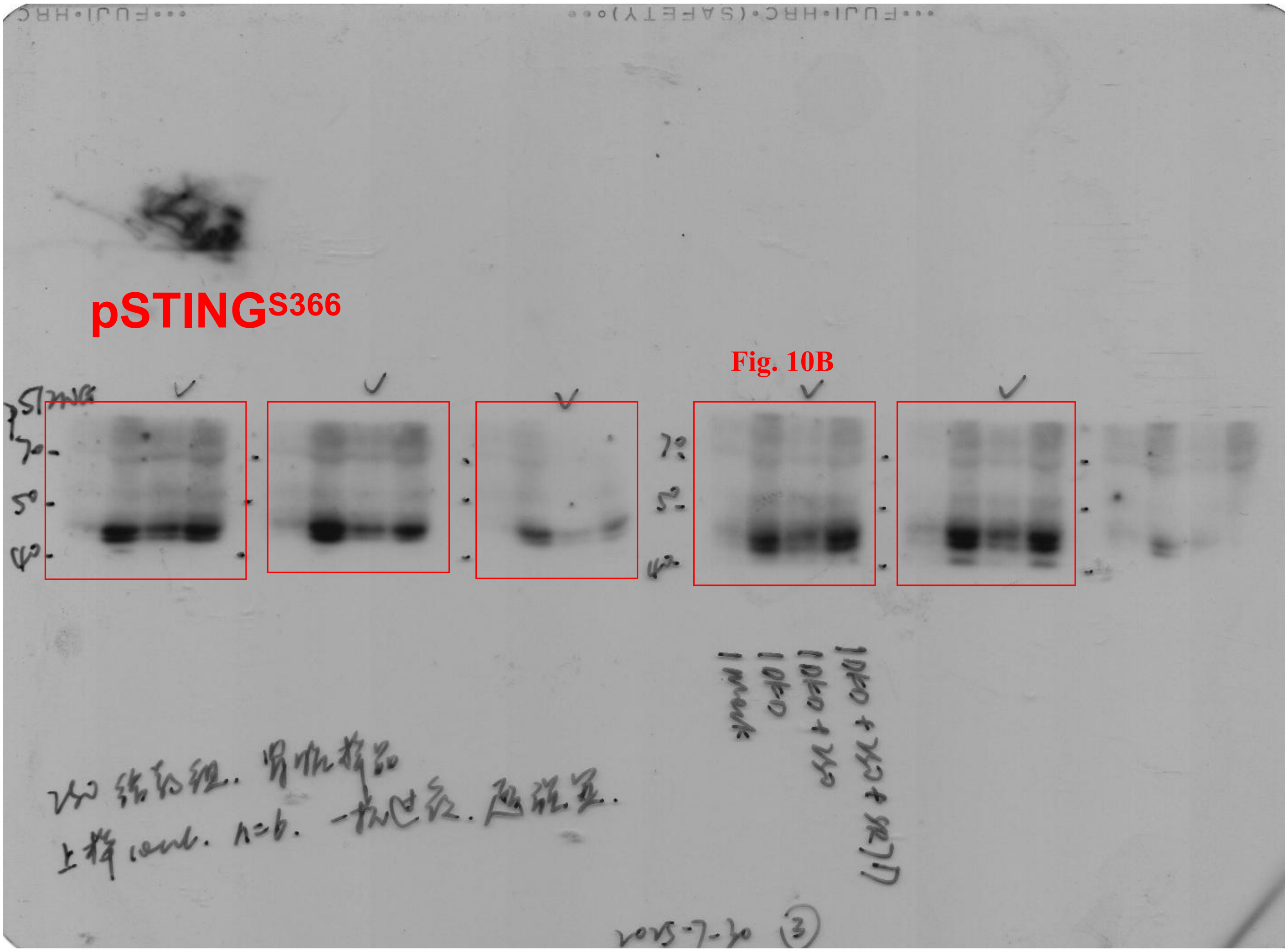
pSTING^{S366}
Fig. 10B

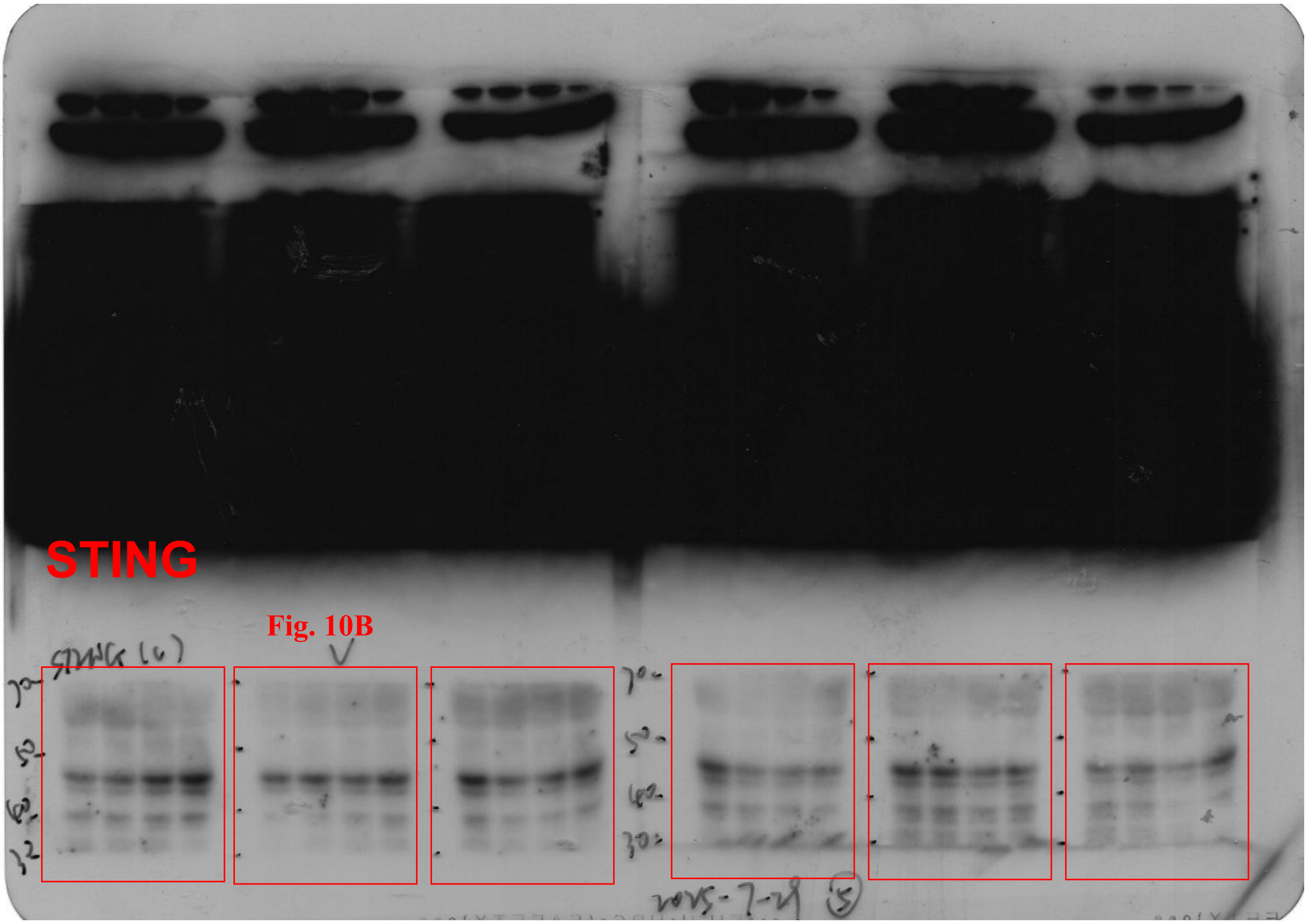
STING
Fig. 10B

pTBK1^{S172}

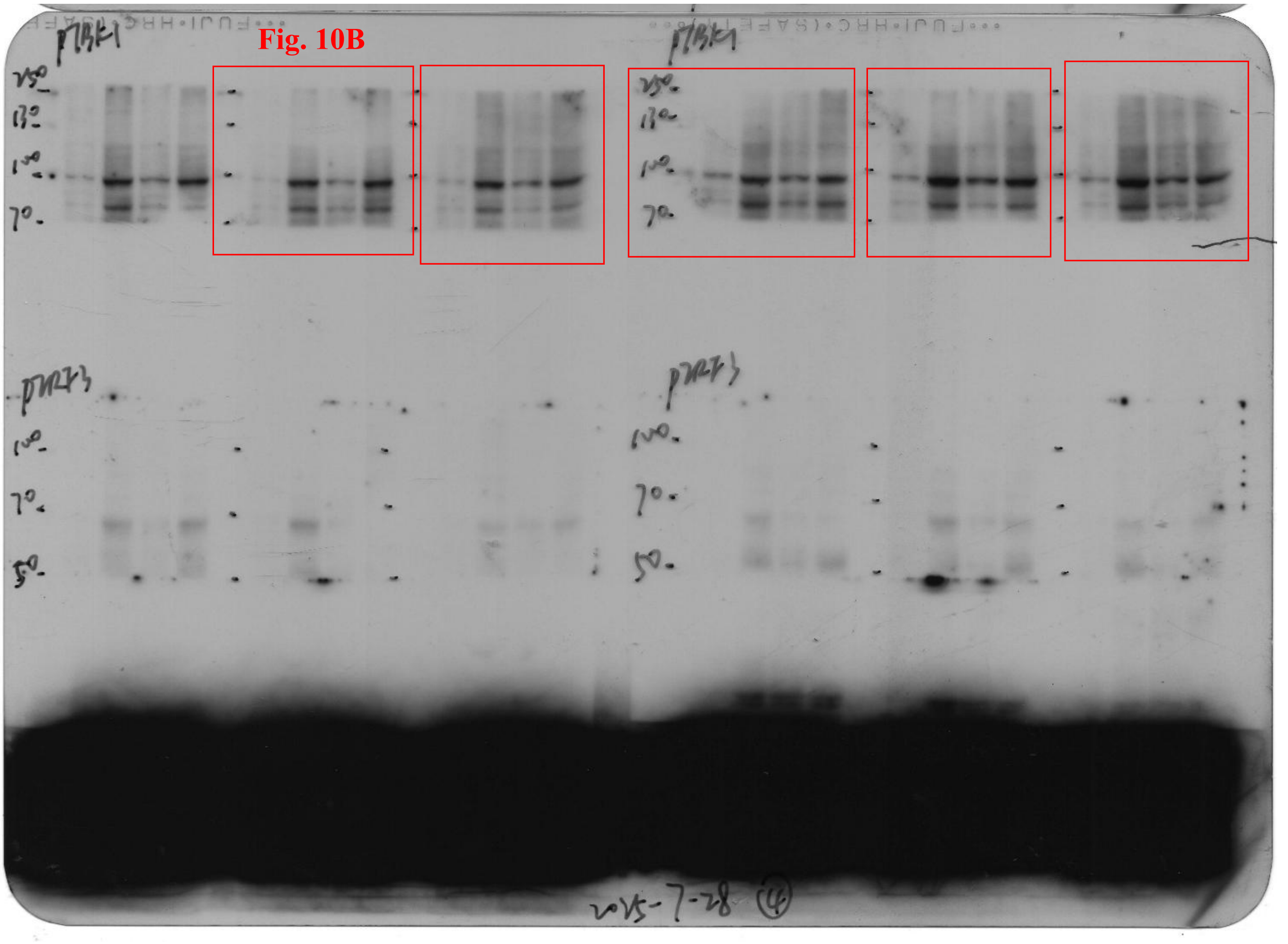

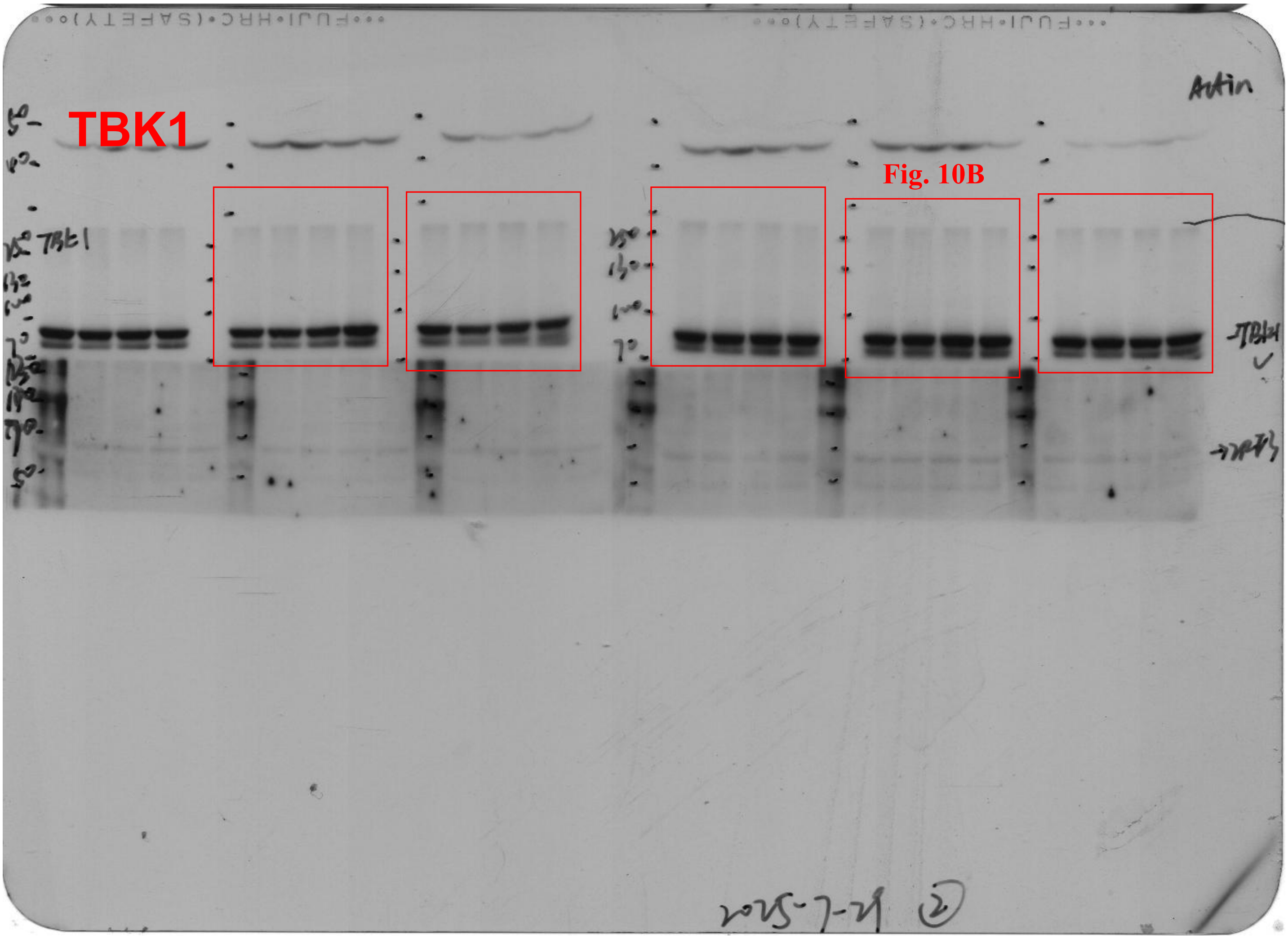
TBK1
Fig. 10B
Actin
TBK1
2025-7-21

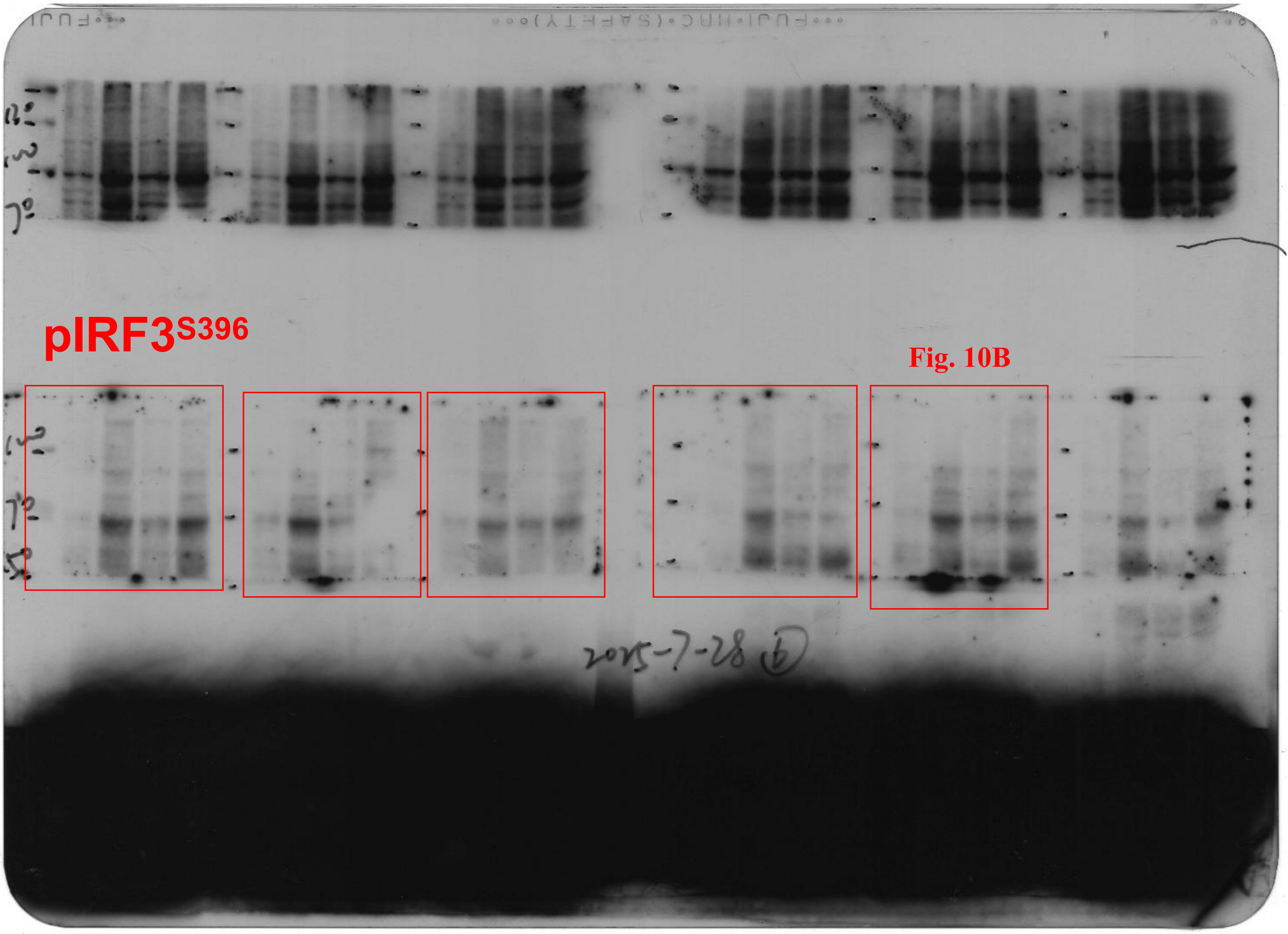
pIRF3S396
Fig. 10B
2015-7-28

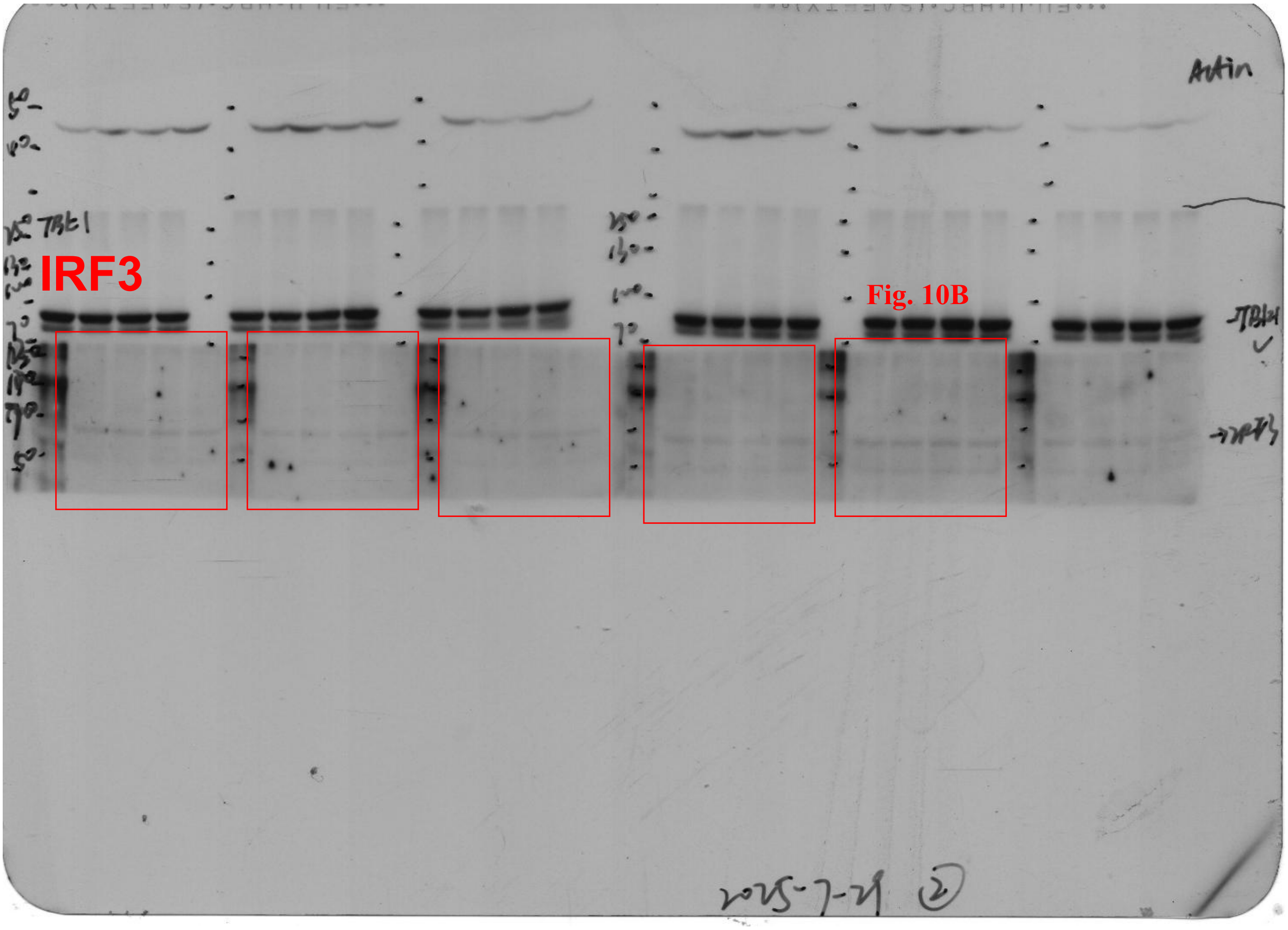

Actin
TBK1
IRF3
Fig. 10B
TBK1
IRF3
2025-7-21 ②

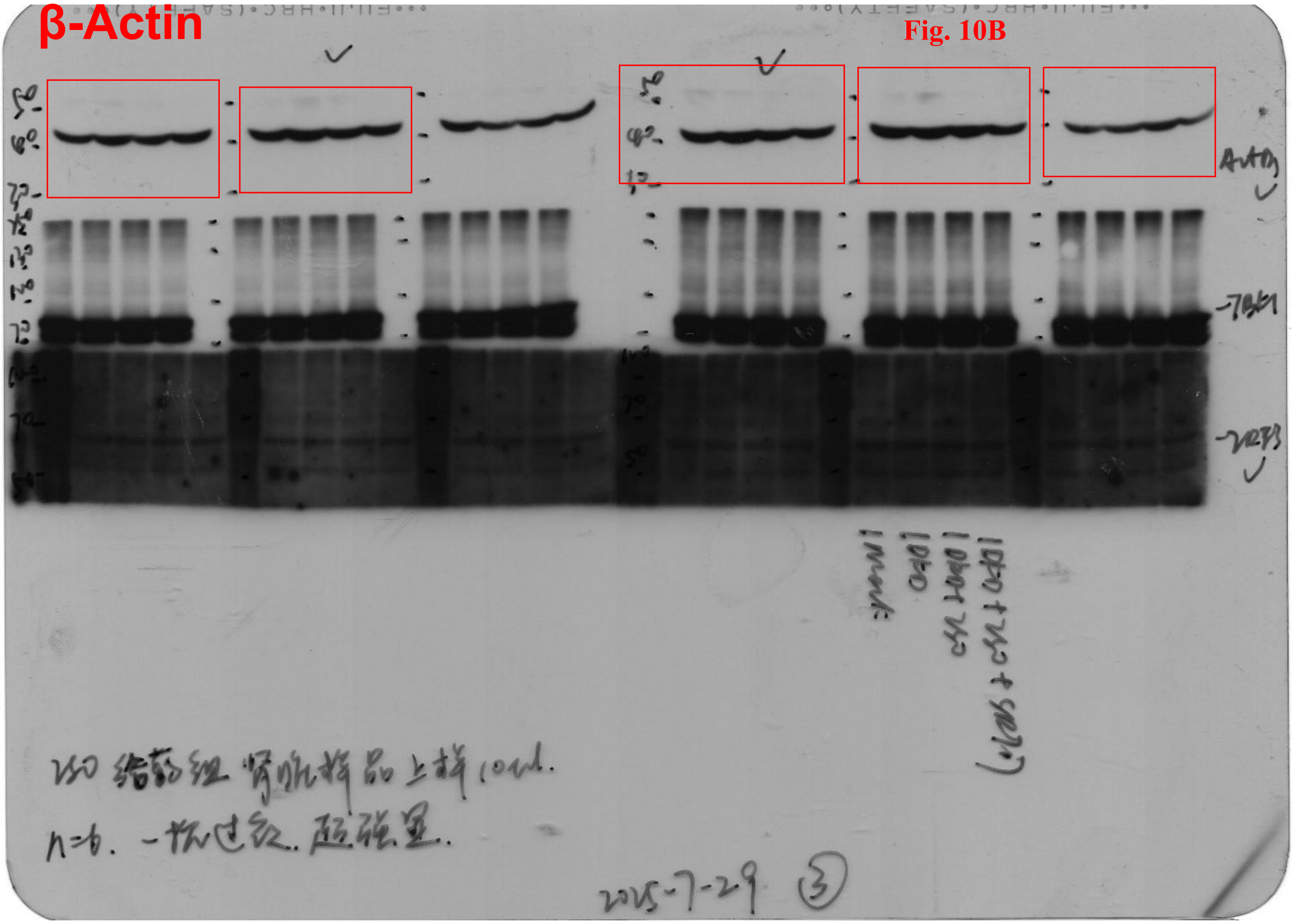
β-Actin
Fig. 10B
Actin
2025-7-29

Figure 10C

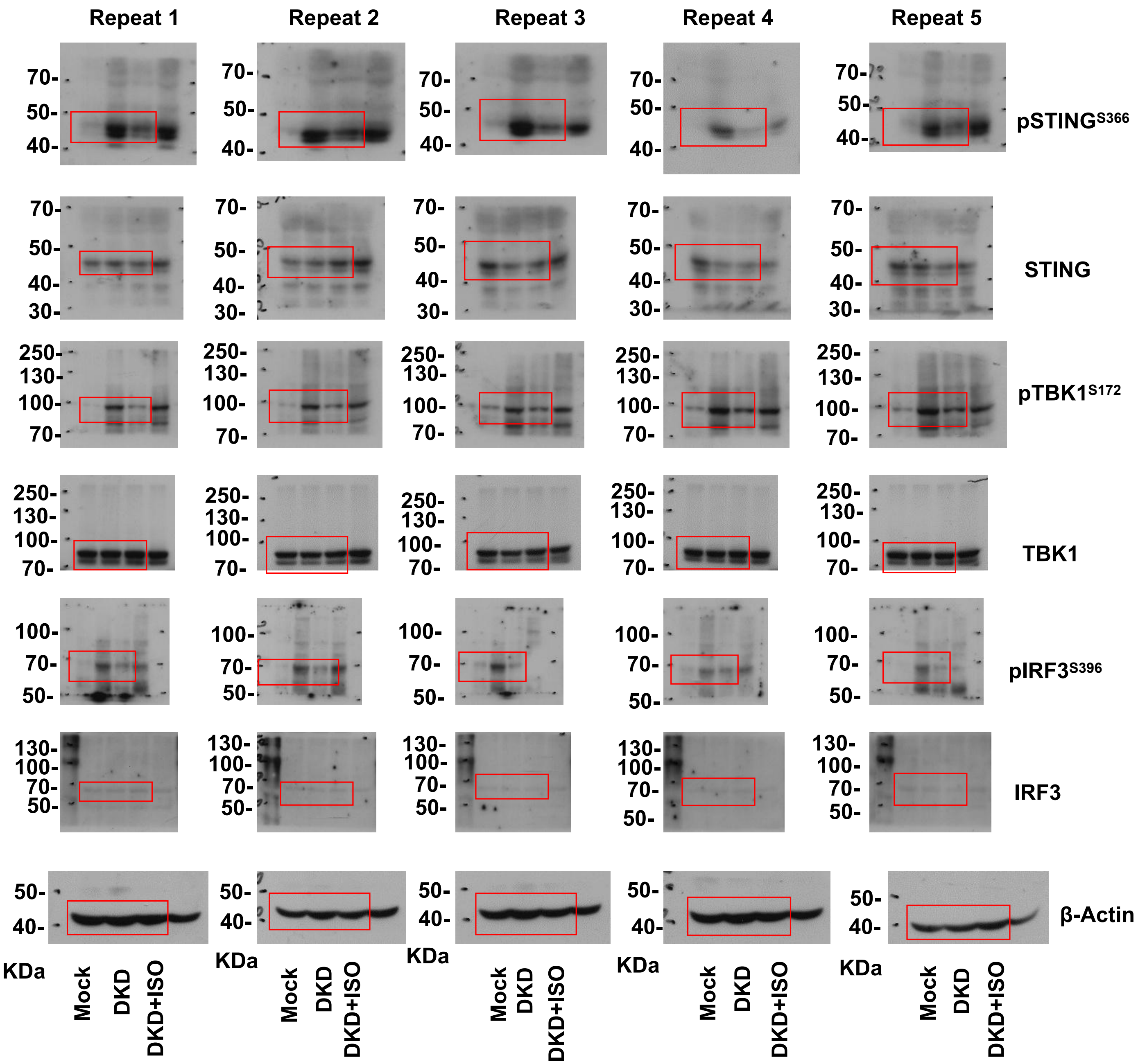

Shown below are the western blot images presented in Figure 10B in the manuscript, along with additional replicate western blot images included in the statistical analysis.
